# Accuracy of Presepsin in Sepsis Diagnosis: A Systematic Review and Meta-Analysis

**DOI:** 10.1371/journal.pone.0133057

**Published:** 2015-07-20

**Authors:** Jiayuan Wu, Liren Hu, Gaohua Zhang, Fenping Wu, Taiping He

**Affiliations:** 1 Nutritional Department, The Affiliated Hospital of Guangdong Medical College, Zhanjiang, Guangdong, China; 2 Department of Epidemiology and Health Statistics, School of Public Health, Guangdong Medical College, Zhanjiang, Guangdong, China; 3 Department of Radiotherapy, The Seventh People's Hospital of Chengdu, The Oncology Hospital of Chengdu, Chengdu, Sichuan Province, China; 4 School of Public Health, Guangdong Medical College, Zhanjiang, Guangdong Province, China; Erasmus Medical Centre, NETHERLANDS

## Abstract

**Objective:**

It’s difficult to differentiate sepsis from non-sepsis, especially non-infectious SIRS, because no good standard exists for proof of infection. Soluble CD14 subtype (sCD14-ST), recently re-named presepsin, was identified as a new marker for the diagnosis of sepsis in several reports. However, the findings were based on the results of individual clinical trials, rather than a comprehensive and overall estimation. Thus, we conducted this systematic review and meta-analysis to estimate the pooled accuracy of presepsin in patients with sepsis suspect.

**Methods:**

A comprehensive electronic search was performed via internet retrieval system up to 15 December 2014. Methodological quality assessment was applied by using the QUADAS2 tool. The diagnostic value of presepsin in sepsis was evaluated by using the pooled estimate of sensitivity, specificity, likelihood ratio, and diagnostic odds ratio, as well as summary receiver operating characteristics curve.

**Results:**

Nine studies with 10 trials and 2159 cases were included in the study. Only two trials had low concerns regarding applicability, whereas all trials were deemed to be at high risk of bias. Heterogeneity existed in the non-threshold effect, but not in the threshold effect. The pooled sensitivity of presepsin for sepsis was 0.78 (0.76–0.80), pooled specificity was 0.83 (0.80–0.85), pooled positive likelihood ratio was 4.63 (3.27–6.55), pooled negative likelihood ratio was 0.22 (0.16–0.30), and pooled diagnostic odds ratio was 21.73 (12.81–36.86). The area under curve of summary receiver operating characteristics curve was 0.89 (95%CI: 0.84 to 0.94) and Q* index was 0.82 (95%CI: 0.77 to 0.87).

**Conclusion:**

This meta-analysis demonstrates that presepsin had some superiority in the management of patients, and may be a helpful and valuable biomarker in early diagnosis of sepsis. However, presepsin showed a moderate diagnostic accuracy in differentiating sepsis from non-sepsis which prevented it from being recommended as a definitive test for diagnosing sepsis in isolation, but the results should be interpreted cautiously.

## Introduction

Sepsis is a type of systematic inflammatory response syndrome (SIRS) caused by the invasion of pathogens or conditional pathogenic bacteria into the blood circulation. It can develop into severe sepsis, septic shock, and multiple organ failure. Sepsis occurs in 1%–2% of all hospitalized patients and accounts for as much as 25% of intensive care unit (ICU) cases [1]. When accompanied by organ system dysfunction or cardiovascular shock, severe sepsis or septic shock occurs and causes millions of deaths worldwide each year [2, 3]. However, there is no good standard exists for proof of infection, no matter blood microbiological cultures which often lead to a late and imprecise report, or clinical symptoms which are non-specific and overlap with signs of SIRS without infection [4]. Delay of diagnosis and treatment with appropriate antimicrobial chemotherapy is the main reason for high morbidity and mortality associated with sepsis, thus looking for a reliable and timely biomarker for sepsis is of utmost importance [5]. At present, more than 178 markers have been found for sepsis, most of which are intermediate products of the inflammatory process and some are sepsis pro-inflammatory cytokines [6]. However, the most reliable biomarkers for precise diagnosis and prediction of the future process of patients suffering from severe sepsis or septic shock are still uncertain or are controversial [7].

As a glycoprotein expressed on monocytes and macrophages, cluster of differentiation 14 (CD14) serves as a receptor of the lipopolysaccharide (LPS)-lipopolysaccharide binding protein complexes and activates a series of signal transduction pathways and inflammatory cascades that finally lead to SIRS [8]. CD14 has two forms, namely, a membrane-bound CD14 (mCD14) and soluble CD14 (sCD14). sCD14 plays an important role in mediating the immune responses to LPS of CD14-negative cells, such as endothelial and epithelial cells. During inflammatory stress, sCD14 is cleaved in plasma, and the N-terminal fragment of 13 kDa has been identified as sCD14 subtype (sCD14-ST; also known as presepsin) [9].

In 2004, the value of presepsin in the diagnosis and evaluation of sepsis was discovered [10], and it has become an alternative biomarker to aid the diagnosis of sepsis. Since then, several studies have reported this compound as a new biomarker in the prediction of sepsis. However, there was a large variability regarding the results and sample sizes of these studies. For instance, the specificity in the report of Palmiere et al. [11] was only 0.44, whereas specificity was 0.98 in the study of Vodnik et al. [12]. Thus, the real value of presepsin in diagnosing sepsis is uncertain. Moreover, the findings of present reports were based on the results of individual clinical trials, and the literature lacks a pooled and robust appraisal of all the evidence for the diagnostic accuracy of presepsin testing. Systematic review and meta-analysis of the diagnostic efficiency are rigorous approaches for examining and synthesizing the evidence in the evaluation of the diagnostic and screening test [13]. Therefore, we conduct this systematic review and meta-analysis to evaluate the relationship between presepsin and sepsis to precisely estimate the diagnostic accuracy of the presepsin test.

## Materials and Methods

### Literature Search

A comprehensive electronic search of the PubMed, Embase, Medline, Cochrane Library, and China National Knowledge Infrastructure (CNKI) was performed via the Internet retrieval system. No language limitation was indicated, and the articles’ inclusion period was until 15 December 2014. Search terms included (“presepsin,” or “sCD14-ST,” or “soluble CD14 subtype,”) and (“sepsis”) and (“diagnosis,” or “diagnostic value,” or “diagnostic biomarker”). In addition, content experts were contacted, and bibliographies of the relevant studies were reviewed to identify additional references. When multiple publications with the same or overlapping patient population from the same institution were identified, only the published report with the largest series was included. This meta-analysis was reported following the Preferred Reporting Items for Systematic Review and Meta-Analysis statement [14].

### Study Selection and Data Extraction

A study was eligible for our meta-analysis if it satisfied the following requirements: (1) its purpose was to evaluate or explore the diagnostic value of presepsin as a single index for differentiation between critically ill patients with sepsis from those of non-infection, such as patients with SIRS without infection, with or without healthy people; (2) data were available for calculating the true positives, false positive, false negatives, and true negatives; (3) applying the clinical criteria of the American College of Chest Physicians and Society of Critical Care Medicine (ACCP/SCCM) [15–17] as reference standard for defining sepsis and SIRS; and (4) with a prospective controlled design. The studies considered ineligible for the meta-analysis were as follows: reviews, conference abstracts, editorials, or case reports; duplicate studies; and those with insufficient information to calculate accuracy estimates.

All data extractions were independently completed by two authors (WJY and HLR) and were checked by a third reviewer (HTP). Any disagreement was resolved through discussion. The following data were extracted from each eligible study: authors, years of publication, locations of the study, selection and characteristics of the sample population, diagnostic test performed, cut-off value, sensitivity, and specificity.

### Quality Assessment

Each full-text article was reviewed independently by two authors (WJY and HLR) and scored with the Quality Assessment of Diagnostic Accuracy Studies 2 (QUADAS-2) tool, and all disagreements were resolved by consensus. QUADAS-2 tool consists of four domains: patient selection, index test, reference standard, and flow/timing. “Risk of bias” was evaluated for all the domains, whereas “concerns regarding applicability” was evaluated for the first three domains. QUADAS-2 does not utilize a comprehensive quality score, but rather an overall judgment of “low,” “high,” or “unclear” risk. For judging the “risk of bias,” the reviewers analyzed all articles by answering each signaling question with a “yes,” “no,” or “unclear” and then by recording the following risk scores for each domain: “L” for “low risk of bias,” “H” for “high risk,” and “U” for “unclear.” In QUADAS-2, “applicability” means whether certain aspects of an individual research match the review’s question or not. The principle and method of judging the “concerns regarding applicability” are the same as those of the “risk of bias,” but without any signaling question. For overall judgment of “low risk of bias” or “low concern regarding applicability,” a study must be ranked “low” on all relevant domains. If a study receives a “high” or “unclear” rating in one or more domains, then it may be judged as “at risk of bias” or having “concerns regarding applicability” [18].

### Statistical Analysis

Data analysis was performed using the Meta-DiSc statistical software version 1.4 [19]. Sensitivity (Se), specificity (Sp), positive likelihood ratio (PLR), negative likelihood ratio (NLR), and diagnostic odds ratio (DOR) were computed for each study. DOR is a comprehensive measure for both Se and Sp or both PLR and NLR, and is regarded as an appropriate global measure for comparing the accuracy of different diagnostic tests, which is estimated by the following formula: (sensitivity/[1-sensitivity])/([1-specificity]/specificity) [20]. Pooled summary effect estimates were calculated using a random effects model (DerSimonian and Laird method) when high heterogeneity exists; otherwise, a fixed effect model (Mantel–Haenszel method) was used [21].

A summary receiver operating characteristics (SROC) curve was drawn to solve the inconsistency of various research results. Serving as global measures for the SROC curve, the area under curve (AUC), which could serve as a probability of correctly recognizing cases and non-cases by a diagnostic test, was also interpreted according to the following guidelines: low for 0.5–0.7, moderate for 0.7–0.9, and high for > 0.9 [22, 23]; as well as the Q* value, which is the point on the SROC curve where sensitivity equals specificity and is in the range 0–1 (1 indicates better test performance) [24, 25].

The heterogeneity of a diagnostic test is caused by threshold or non-threshold effects in general. In this study, the heterogeneity caused by threshold effect was evaluated by the appearance of an SROC curve (a “shoulder-like” distribution suggested the existence of a threshold effect) and the calculation of Spearman correlation coefficient (*ρ*) between sensitivity logarithm and (1-specificity) logarithms. Furthermore, heterogeneity caused by non-threshold effect was quantified by applying the *χ* ^*2*^ (for Se and Sp) and the Cochrane-*Q* test (for PLR, NLR, and DOR) and by determining the *I* ^2^ metric. Statistical significance was set at *P* < 0.05 or *I* ^2^ > 50% for the heterogeneity testing. A meta-regression was also performed to determine the factors for heterogeneity that have been influenced by the non-threshold effect. To eliminate the influence of the confounding factors, we also conducted a sensitivity analysis by calculating the pooled DOR and 95% CI after omitting the studies which including some possible confounding factors. Funnel plots, Begg’s rank correlation, and Egger’s linear regression method were also conducted to evaluate the potential publication bias through the STATA version 11.0 (STATA Corporation, College Station, TX, USA) [26, 27].

## Results

### Characteristics of included studies

Nine studies [7, 12, 28–34] met the inclusion criteria for our meta-analysis, one [34] of which included two trials. Therefore, a total of 10 trials with 2159 cases were included (1320 patients with sepsis, 512 with SIRS of non-infectious origin, and 327 healthy people), the sample sizes of which ranged from 104 to 959. These 10 trials were eligible for this meta-analysis. These studies principally originated from Western Europe (2 from Italy [29, 33], and 1 from Germany [7]) and Eastern Asia (3 from China [30–32], 1 from Korea [28], and 1 from Japan [34]). These studies along with one study from Serbia [12] were published between 2011 and 2014. Seven studies were written in English [7, 12, 28, 29, 32–34], and the other two were written in Chinese [30, 31]. All of the 9 studies were conducted in a prospective controlled design. Three studies were done in internal care unit (ICU) or critical care unit (CCU) [7, 29, 31], five studies in emergency department (ED) [12, 28, 31, 32, 33], while only one in CCU and ED [34]. Four studies clearly stated that the blinding method was used in presepsin determination [7, 28, 31, 33], and three studies included only patients with non-infectious SIRS as controls [31, 33, 34], while the other six studies included both patients of SIRS without infection and healthy people in the control group [7, 12, 28–30, 32].

**Fig 1:**
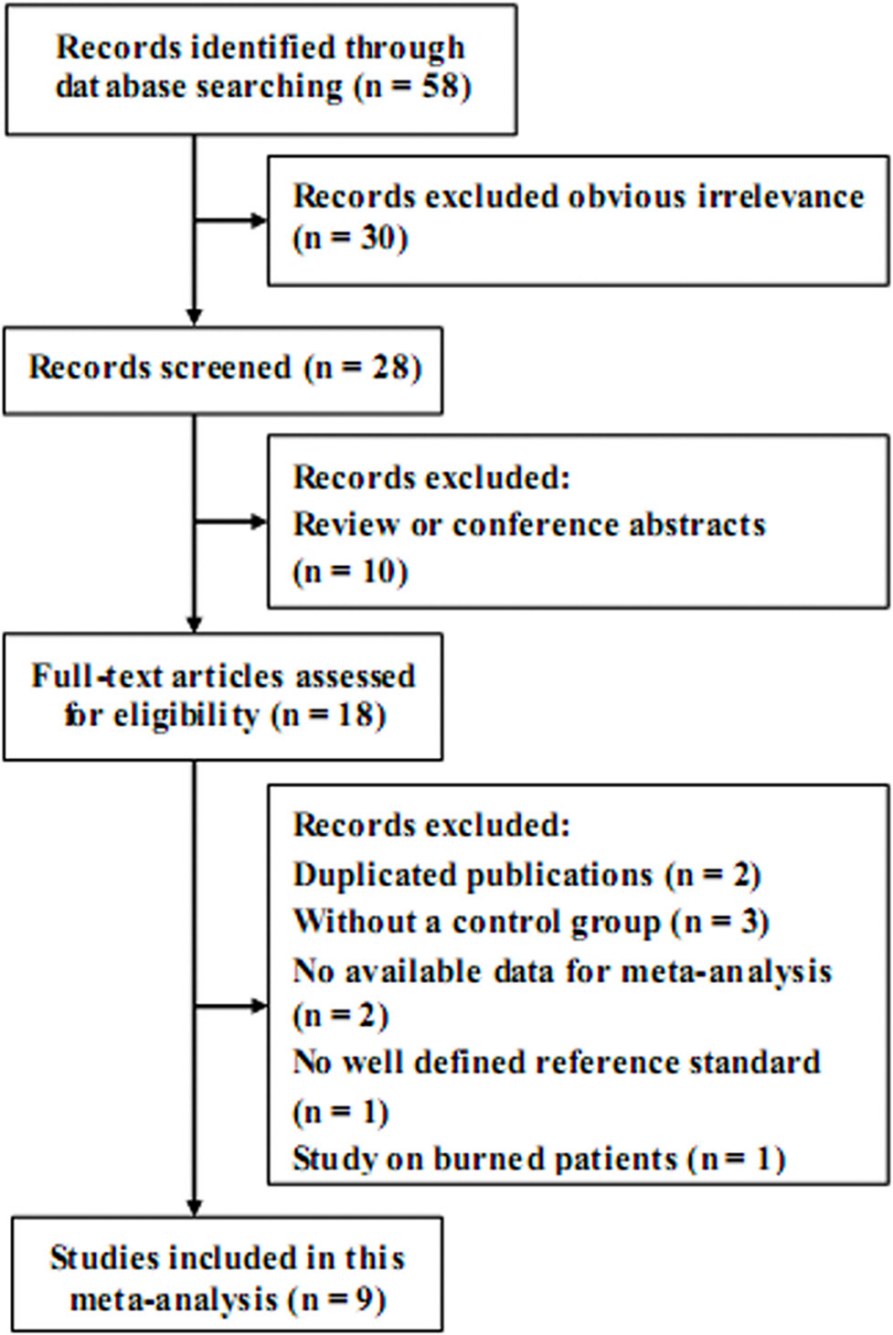
Flow diagram of the study selection process and specific reasons for exclusion in the meta-analysis.

A summary of the characteristics of the 9 studies is outlined in Table 1, and the clinical natures of each trial are shown in Table 2. A flow diagram describes the details of the study selection progress (Fig 1).

**Table 1:** Characteristics of the included studies.

Author (year)	Nation	Language	Recruitment time	n	Male/female	T/C	Testing method	Blind
Behnes M (2014) [7]	Germany	English	Since 2011.10	176	111/65	107/69	CLEIA	Yes
Kweon OJ (2014) [28]	Korea	English	2012.9~2013.7	118	59/59	73/45	CLEIA	Yes
Sargentini V (2014) [29]	Italy	English	2013.3~2013.7	104	47/57	60/44	CLEIA	NR
Su MH (2014) [30]	China	Chinese	2012.11~2013.8	115	65/50	72/43	CLEIA	NR
Yu J (2014) [31]	China	Chinese	2012.6~2012.12	176	119/57	63/113	CLEIA	Yes
Liu B (2013) [32]	China	English	2011.12~2012.10	959	570/389	680/279	CLEIA	NR
Ulla M (2013) [33]	Italy	English	2012.1~2013.1	189	116/73	106/83	CLEIA	Yes
Vodnik T (2013) [12]	Serbia	English	NR	130	71/59	30/100	CLEIA	NR
Shozushima T (2011) [34]	Japan	English	2009.8~2010.7	192	117/75	129/63	CLEIA	NR

NR: none reported; T/C: test group/control group; CLEIA: chemiluminescent enzyme immunoassay.

**Table 2:** Clinical nature of the included studies.

Author (year)	Setting	Admission category	Severity	Controls	Sampling time
Behnes M (2014) [7]	ICU	Medical	Sepsis, severe sepsis, sepsis shock	SIRS without infection, health	At clinical onset
Kweon OJ(2014) [28]	ED	Medical	Sepsis, severe sepsis, sepsis shock	SIRS without infection, health	At admission
Sargentini V (2014) [29]	CCU	Medical, traumatic	Sepsis, severe sepsis	SIRS without infection, health	At admission
Su MH (2014) [30]	ED	Medical	Sepsis, severe sepsis, sepsis shock	SIRS without infection, health	Before any treatment
Yu J (2014) [31]	ICU	Traumatic	Sepsis	SIRS without infection	At clinical onset
Liu B (2013) [32]	ED	Medical	Sepsis, severe sepsis, sepsis shock	SIRS without infection, health	At admission
Ulla M (2013) [33]	ED	Medical, surgical, traumatic	Sepsis, severe sepsis,sepsis shock	SIRS without infection	At first medical evaluation
Vodnik T (2013) [12]	ED	Surgical	Sepsis, severe sepsis, sepsis shock	SIRS without infection, health	At admission
Shozushima T (2011) [34]	CCU, ED	Medical, traumatic	Sepsis, severe sepsis	SIRS without infection	At admission

NR: none reported; ICU: internal care unit; ED: emergency department; CCU: critical care unit; SIRS: systemic inflammatory response syndrome; ACCP/SCCM: the American College of Chest Physicians/Society of Critical Care Medicine Consensus Conference

### Results of quality assessment

The results of the quality assessment are listed in Table 3, and the details are presented in S1 Table. When the QUADAS-2 tool was used to assess the risk of bias in the 10 trials, 7 trials showed problems in patient selection, 5 showed problems in reference standard, 4 showed problems in study flow, and all 10 trials showed problems in the index test. Moreover, when the concerns of applicability were considered, 8 trials showed problems in patient selection, but the reference standard and index test were used appropriately in all trials.

**Table 3:** Results of QUADAS-2 quality assessment for each trial.

Studies	Risk of bias					Concerns of applicability			
	Patient selection	Index test	Reference standard	Flow and time	Overall	Patient	Index test	Reference standard	Overall
Behnes M (2014) [7]	?	?	☺	☺	High	☹	☺	☺	High
Kweon OJ (2014) [28]	☺	?	☺	☺	High	☹	☺	☺	High
Sargentini V (2014) [29]	☺	?	?	☺	High	☹	☺	☺	High
Su MH (2014) [30]	?	?	☺	?	High	☹	☺	☺	High
Yu J (2014) [31]	☺	?	☺	☺	High	☺	☺	☺	Low
Liu B (2013) [32]	?	☹	?	?	High	☹	☺	☺	High
Ulla M (2013) [33]	?	?	☺	?	High	☺	☺	☺	Low
Vodnik T (2013) [12]	☹	☹	?	?	High	☹	☺	☺	High
Shozushima T (2011)^a^ [34]	?	?	?	☺	High	☹	☺	☺	High
Shozushima T (2011)^b^ [34]	?	?	?	☺	High	☹	☺	☺	High

^a^ Results of first of two trials in this article

^b^ Results of second of two trials in this article

☺ low risk; ☹ high risk;? unclear risk.

In general, all trials were at risk of bias. Two trials had low concerns regarding applicability, whereas the other 8 trials showed problems regarding applicability.

### Results of heterogeneity test

**Fig 2:**
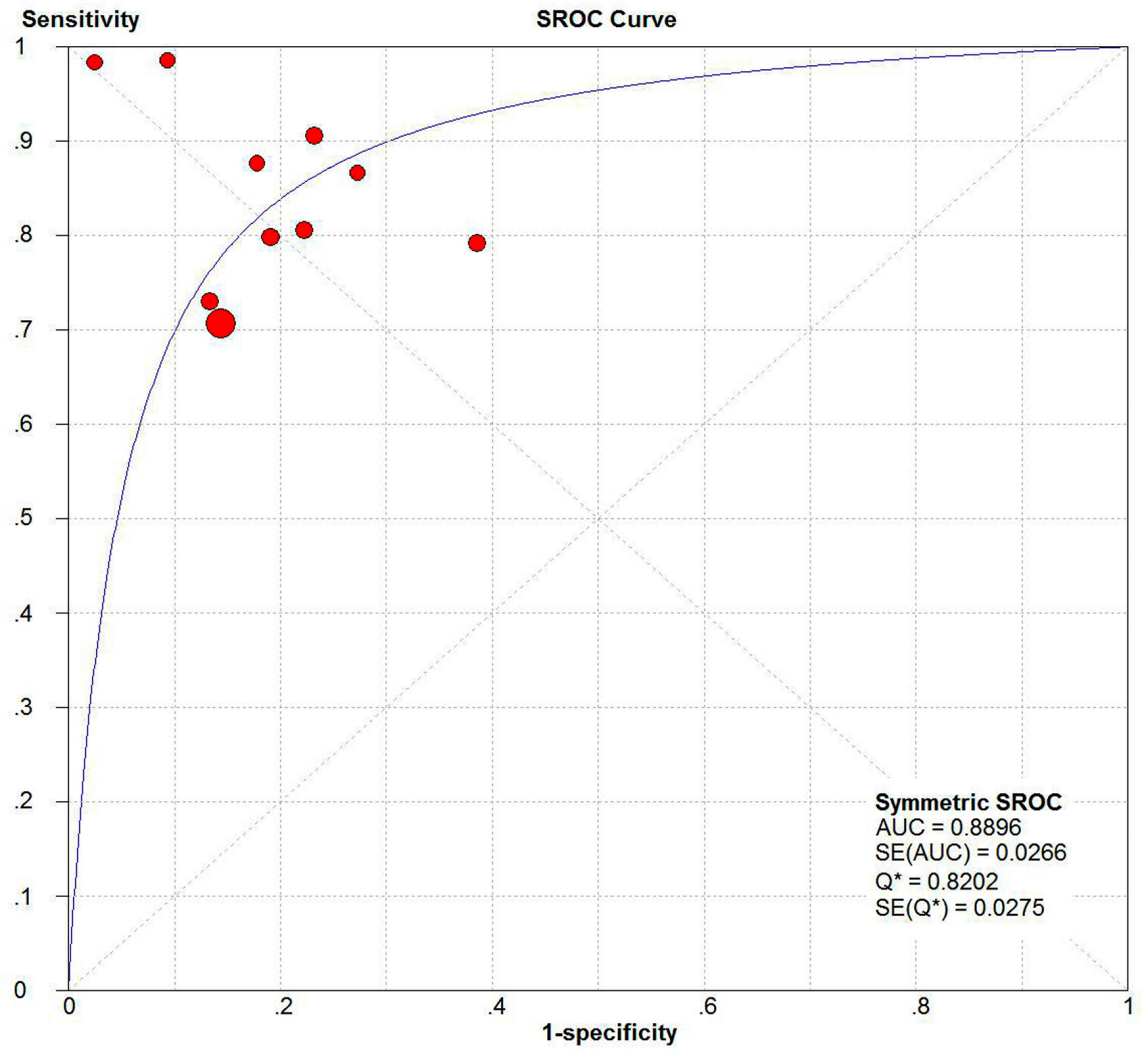
Summary receiver operator characteristic plots with 95% CIs of sensitivity against (1-specificity) of presepsin testing for sepsis.

From the appearance of the SROC curve (Fig 2) and the estimation of the Spearman correlation coefficient (*ρ* = –0.261, *P* = 0.467), we could conclude that no threshold effect existed.

However, when non-threshold effect was investigated, significant heterogeneity was measured in the overall Se (*χ* ^*2*^ = 83.47, *P* < 0.001, *I* ^2^ = 89.2%), Sp (*χ* ^*2*^ = 56.40, *P* < 0.001, *I* ^2^ = 84.0%), PLR (Cochran-*Q* = 46.13, *P* < 0.001, *I* ^2^ = 80.5%), NLR (Cochran-*Q* = 45.49, *P* < 0.001, *I* ^2^ = 80.2%), and DOR (Cochran-*Q* = 35.15, *P* = 0.0001, *I* ^2^ = 74.4%). Therefore, a random-effect model was applied for data synthesis. To determine the sources of heterogeneity, we used location, number of cases, blind, cutoff value, setting, admission category, and control’s component as variables in the meta-regression analysis. The results are shown in Table 4. Results showed that the heterogeneity could not be explained by meta regression analysis.

**Table 4:** Possible sources of heterogeneity of meta-analysis (Results of meta-regression analysis).

Variance	Coefficient	Standard error	*P* value	RDOR (95% *CI*)
Location (Europe vs. Asia)	2.341	0.8083	0.0626	10.40 (0.79; 136.26)
No. of cases (> 150 vs. < 150)	- 0.601	0.8330	0.5227	0.55 (0.04; 7.77)
Cut-off value (≥ 600 pg/ml vs. < 600 pg/ml)	0.628	1.0447	0.5698	1.87 (0.15; 24.15)
Blind (Blind vs. NR)	0.651	0.8551	0.4756	1.92 (0.24; 15.53)
Setting (ICU/CCU vs. ED vs. CCU + ED)	- 0.533	0.2762	0.1494	0.59 (0.24; 1.41)
Admission category (only medical vs. others)	0.983	0.9208	0.3642	2.67 (0.14; 50.04)
Controls (SIRS vs. SIRS + healthy)	1.555	1.0332	0.2294	4.73 (0.18; 126.84)

RDOR: relative diagnostic odds ratio; CI: confidence interval; NR: none reported; ICU: internal care unit; ED: emergency department; CCU: critical care unit; SIRS: systemic inflammatory response syndrome.

### Results of meta-analysis

For the 9 studies that represented 10 trials, cutoff values were ranged from 317 pg/ml to 700 pg/ml in each trial. The reported sensitivity of presepsin diagnosing sepsis ranged from 0.71 to 1.00, the specificity ranged from 0.62 to 0.98, the positive likelihood ratio ranged from 1.71 to 39.75, the negative likelihood ratio ranged from 0.02 to 0.34, and the diagnostic odds ratio ranged from 6.09 to 2403.40.

**Fig 3:**
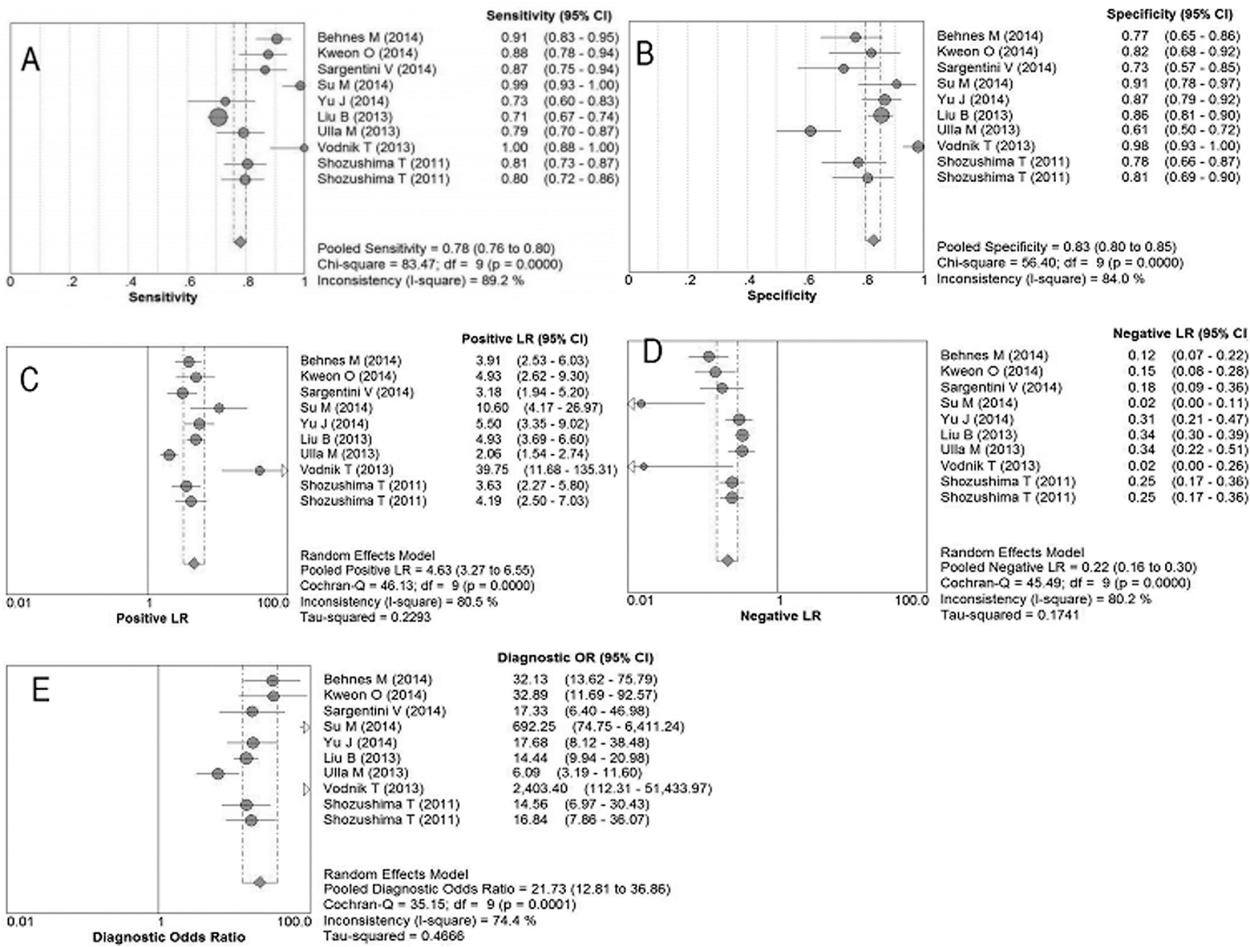
Forest plots of the pooled results for presepsin in diagnosing sepsis. Each circle shows the point estimate of the outcome from each included trial. (A) Forrest plot to assess the pooled sensitivity. (B) Forrest plot to assess the pooled specificity. (C) Forrest plot to assess the pooled positive likelihood ratio. (D) Forrest plot to assess the pooled negative likelihood ratio. (E) Forrest plot to assess the pooled diagnostic odds ratio.

Overall, the pooled Se of presepsin for sepsis was 0.78 (95%CI: 0.76 to 0.80) (Fig 3A), pooled Sp was 0.83 (95%CI: 0.80 to 0.85) (Fig 3B), pooled PLR was 4.63 (95%CI: 3.27 to 6.55) (Fig 3C), pooled NLR was 0.22 (95%CI: 0.16 to 0.30) (Fig 3D), and pooled DOR was 21.73 (95%CI: 12.81 to 36.86) (Fig 3E). In addition, the AUC of SROC curve was 0.89 (95%CI: 0.84 to 0.94), and Q* index was 0.82 (95%CI: 0.77 to 0.87) (Fig 2).

The results of each trial, and overall outcomes were listed in Table 5.

**Table 5:** The diagnostic parameters of presepsin for sepsis in the included trials, and overall outcome.

Study	Cut-off (pg/ml)	TP	FP	FN	TN	Se	Sp	PLR	NLR	DOR
Behnes (2014) [7]	700	97	16	10	53	0.91 (0.83–0.95)	0.77 (0.65–0.86)	3.91 (2.53–6.03)	0.12 (0.07–0.22)	32.13 (13.62–75.79)
Kweon (2014) [28]	430	64	8	9	37	0.88 (0.78–0.94)	0.82 (0.68–0.92)	4.93 (2.62–9.30)	0.15 (0.08–0.28)	32.89 (11.69–92.57)
Sargentini (2014) [29]	600	52	12	8	32	0.87 (0.75–0.94)	0.73 (0.57–0.85)	3.18 (1.94–5.20)	0.18 (0.09–0.36)	17.33 (6.40–46.98)
Su M (2014) [30]	407	71	4	1	39	0.99 (0.93–1.00)	0.91 (0.78–0.97)	10.60 (4.17–26.97)	0.02 (0.00–0.11)	692.25 (74.75–6411.24)
Yu J (2014) [31]	540	46	15	17	98	0.73 (0.60–0.83)	0.87 (0.79–0.92)	5.50 (3.35–9.02)	0.31 (0.21–0.47)	17.68 (8.12–38.48)
Liu B (2013) [32]	317	481	40	199	239	0.71 (0.67–0.74)	0.86 (0.81–0.90)	4.93 (3.69–6.60)	0.34 (0.30–0.39)	14.44 (9.94–20.98)
Ulla M (2013) [33]	600	84	32	22	51	0.79 (0.70–0.87)	0.61 (0.50–0.72)	2.06 (1.54–2.74)	0.34 (0.22–0.51)	6.09 (3.19–11.60)
Vodnik T (2013) [12]	630	30	2	0	98	1.00 (0.88–1.00)	0.98 (0.93–1.00)	39.75 (11.68–135.31)	0.02 (0.00–0.26)	2403.40 (112.31–51433.97)
Shozushima T (2011) ^a^ [34]	399	104	14	25	49	0.81 (0.73–0.87)	0.78 (0.66–0.87)	3.63 (2.27–5.80)	0.25 (0.17–0.36)	14.56 (6.97–30.43)
Shozushima T (2011) ^b^ [34]	415	103	12	26	51	0.80 (0.72–0.86)	0.81 (0.69–0.90)	4.19 (2.50–7.03)	0.25 (0.17–0.36)	16.84 (7.86–36.07)
Overall outcome						0.78 (0.76–0.80)	0.83 (0.80–0.85)	4.63 (3.27–6.55)	0.22 (0.16–0.30)	21.73 (12.81–36.86)

^a^ Results of first of two trials in this article

^b^ Results of second of two trials in this article

TP: true positive; FP: false positive; FN: false negative; TN: true negative; Se: sensitivity; Sp: specificity; PLR: positive likelihood ratio; NLR: negative likelihood ratio; DOR: diagnostic odds ratio.

### Results of sensitivity analysis and publication bias

In the sensitivity analysis, the influence of each study on the pooled AUC was examined by omitting each study one at a time. Regardless which study was removed, the pooled AUC estimated by the remaining studies did not change significantly. This result indicated that no individual study dominated the results of the meta-analysis, thereby validating the credibility of the outcomes (Table 6). To compare the diagnostic accuracy of presepsin for sepsis in studies with or without healthy controls, we also conducted a sensitivity analysis by obtaining data from 10 trials (6 trials provided data for controls with healthy people, and 4 provided data for controls without healthy people). When healthy people were included in controls with SIRS of non-infectious origin, the diagnostic accuracy was similar to that of including only SIRS without infection in controls as evaluated by AUC (0.90 [95% CI: 0.88 to 0.92] vs. 0.85 [95%CI: 0.82 to 0.88]; not tested for significance).

**Table 6:** The pooled AUC and 95% CI after omitting each trial in the meta-analysis (The results of sensitivity analysis).

Study	AUC	95% CI
Behnes M (2014)	0.89	0.83–0.95
Kweon OJ (2014)	0.89	0.83–0.95
Sargentini V (2014)	0.90	0.84–0.96
Su MH (2014)	0.88	0.84–0.92
Yu J (2014)	0.89	0.82–0.96
Liu B (2013)	0.89	0.81–0.97
Ulla M (2013)	0.89	0.87–0.91
Vodnik T (2013)	0.88	0.84–0.92
Shozushima T (2011)^a^	0.90	0.84–0.96
Shozushima T (2011)^b^	0.89	0.83–0.95

^a^ Results of first of two trials in this article

^b^ Results of second of two trials in this article

AUC: the area under the summary receiver operating characteristic curve; CI: confidence interval.

**Fig 4:**
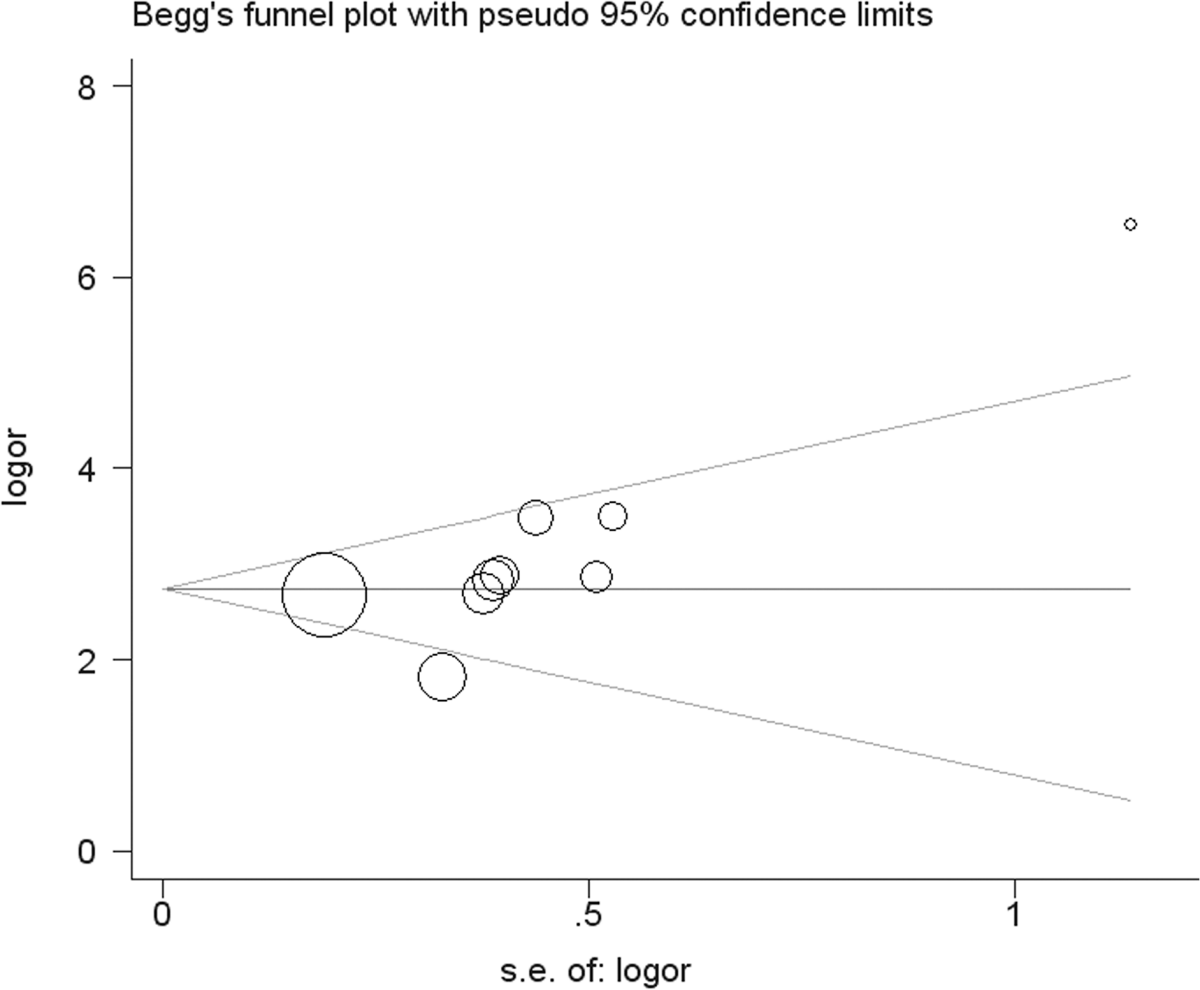
Begg’s funnel plot of publication bias. Each point represents a separate study.

Publication bias was analyzed by funnel plot, Egger’s test, and Begg’s test, which provided negligible evidence of publication bias for the outcome of DOR (*P* value for Egger’s test = 0.140; *P* value for Begg’s test = 0.067). The funnel plot is shown in Fig 4.

## Discussion

At present, various biomarkers (alone or in combination) are used in the diagnosis of sepsis, including procalcitonin (PCT), C-reactive protein (CRP), interleukin (IL), and soluble form of triggering receptor expressed on myeloid cells-1 (Strem-1). However, the clinical value of these biomarkers is still controversy. Moreover, blood culture is treated as the gold criteria for sepsis diagnosis, but it always takes 48–72 hours to obtain the outcome when this approach is used. Blood culture has a low positive rate, which results in diagnosis delay, and the best treatment time is missed. Therefore, finding a reliable biomarker for the early and rapid diagnosis of sepsis is critical.

A large number of studies have found that sCD14 has important pathophysiological implications in the occurrence and development of many human diseases. sCD14 exists in the blood and urine of humans, and comprises 99% of the total amount of CD14 in the human body, with a normal concentration of 2 to 6 μg/ml in serum [35]. sCD14 concentration is closely related to the level of endotoxin and plays a critical role on mediating the inflammatory reaction of endothelial and epithelial cells. As a subtype of sCD14, presepsin can signal a much earlier and faster increase of sepsis when compared with PCT. Hence, presepsin may be a useful diagnostic biomarker for sepsis. However, the results of the current studies on presepsin showed considerable differences, such as in the SE range (0.73–1.00) and in the SP range (0.44–0.98), which may be due to the differences in the sample populations and study designs, differences in the selection of people for the control, and differences in cut-off values. Therefore, a meta-analysis, which is useful in integrating results from independent studies for a specified outcome, was conducted to determine the pooled outcomes.

In our meta-analysis, 9 studies that represented 10 trials were included, of which the sample populations were collected from ICU, ED, and CCU, or were typically seen in medical, surgical, and traumatic. The findings of this meta-analysis were therefore applicable to common clinical settings in which critically ill patients were managed. In these studies, the diagnostic value of presepsin was evaluated by testing the level in blood sample of septic patients compared with that of patients with non sepsis. Blood samples were drawn at admission, or clinical onset, or before any treatment, and presepsin levels were measured by using a chemiluminescent enzyme immunoassay that allowed making automated measurements in a short time. The pooled results indicated that presepsin showed a moderate diagnostic value for distinguishing sepsis from non sepsis. However, systematic reviews of diagnostic accuracy studies are often characterized by a notable heterogeneity caused by the differences in the design and implementation of the studies. Thus, our results should be interpreted cautiously [36].

First, non-infectious SIRS patients were assessed as controls in each study, which is difficult to distinguish from sepsis because the clinical signs often overlap between them. However, healthy people also served as controls in some included studies, which may make the pooled outcomes much higher than the real results. Then we conducted a sensitivity analysis to make an exact evaluation, but the results had not changed much in the index of AUC compared with the overall AUC (0.89 [95%CI: 0.84 to 0.94] vs. 0.85 [95%CI: 0.82 to 0.88]), thus, the including of healthy people seemed to have little influence on the overall outcomes. But further study designed with better homogeneity is advisable.

Second, the cutoff values of presepsin among these included studies varied greatly, even though using the same test method. The difference may lie in the admission category, number of cases, and sample timing. Therefore, this research was limited in terms of qualitative analysis, but not in terms of quantitative analysis.

Third, all included studies declared that infection was confirmed by microbiologically or clinically according to an internationally recognized gold standard, but most failed to provide the information about whether previous antibacterial treatment was used. Under the impact of antibiotic drugs, bacteraemia occurs in only 30% of patients with sepsis [37, 38]. Moreover, the level of presepsin decreased rapidly after treatment [28]. Thus, the absence of such therapeutic details may increase interobserver variability, and add false judgment about the patient’s medical condition [4].

Fourth, extreme heterogeneity existed among these included studies. We detected substantial heterogeneity by bringing the study characteristics and clinical natures into meta-regression analysis, but none of them was responsible for the majority of heterogeneity. Thus, some unrecorded difference may contribute to the heterogeneity, and a study with better design and more homogenous population is needed to avoid the heterogeneity.

Fifth, LPS is a component of the Gram-negative bacterial cell wall. As a receptor of LPS, presepsin is easy to imagine that whether it only work for Gram-negative infections. Studies have shown that the Se of presepsin was not significantly different between Gram-positive and Gram-negative bacterial infections [17, 39, 40], while no disparity of presepsin concentration was found among the infection caused by Gram-positive or Gram-negative bacterial infections [30]. So it seems to be lack of evidence to prove that presepsin is not a predicator only for sepsis induced by Gram-negative cocci infection.

In previous clinical practices, many biomarkers were widely applied in diagnosis of sepsis, and PCT is the most widely used one. Several evidence-based researches have proved that PCT had a low diagnostic performance in differentiating sepsis from critically ill patients, and can not be recommended as the single definitive test for sepsis diagnosis [4, 41, 42]. When compared to PCT, presepsin also showed a similar diagnostic accuracy for sepsis with respect to Se (0.78 [95% CI: 0.76 to 0.80] vs. 0.77 [95% CI: 0.72 to 0.81]), Sp (0.83 [95% CI: 0.80 to 0.85] vs. 0.79 [95% CI: 0.74 to 0.84]), AUC (0.89 [95% CI: 0.84 to 0.94] vs. 0.85 [95% CI: 0.81 to 0.88]), according to a latest evidence about the accuracy of PCT for differentiating sepsis from SIRS without infection [4]. To avoid the bias of results raised by including healthy people in the research, we conducted a sensitivity analysis to exclude these studies. From the results of sensitivity analysis, we noted that, when focused on studies aimed at discriminating sepsis from non-infectious SIRS, the accuracy of presepsin was the same to that of PCT, with respect to AUC (0.85 [95% CI: 0.82 to 0.88] vs. 0.85 [95% CI: 0.81 to 0.88]). Whether a marker can be used alone for the diagnosis of sepsis depends on its diagnostic performance. Therefore, presepsin did not show any advantage in diagnostic performance of sepsis compared to PCT, and it cannot be recommended as a single definitive test for sepsis diagnosis. However, presepsin showed some superiority in the management of patients, such as both presepsin and PCT are elevated in non-infectious SIRS and sepsis, but presepsin can signal a much earlier and faster increase in sepsis, and perform a unique capacity of distinguishing the severity of sepsis [7, 12, 33]. Furthermore, by using the PATHFAST analysis system, presepsin test only takes 17 min and can be conducted at bedside, thereby complying with the guidelines for the diagnosis and treatment of sepsis [1]. Consequently, although presepsin showed a moderate diagnostic accuracy which was similar to PCT, but it was still a helpful biomarker for early diagnosis of sepsis and severity evaluation.

In addition, other limitations are present. First, the search range was limited in published studies, which means we might have missed some unpublished, but valuable studies. Second, diagnostic tests were generally designed in case-control study or cross-sectional study, which belonged to the third level design in evidence-based medicine. Thus, many studies in this area were not designed with a blind method or did not clearly use the blind method to strengthen the argument, which may have contributed to the possibility of bias [43, 44]. Third, this systemic review did not investigate the prognostic value of presepsin in sepsis because not enough evidence is available.

In conclusion, presepsin had some superiority in the management of patients, and may be a helpful and valuable biomarker in early diagnosis of sepsis. However, presepsin showed a moderate diagnostic accuracy in differentiating sepsis from non-sepsis which prevented it from being recommended as a definitive test for diagnosing sepsis in isolation, but the results should be interpreted cautiously due to the heavy heterogeneity and different clinical natures in these included studies. Moreover, further randomized controlled researches are required in the context with unified clinical information.

## Supporting Information

S1 Checklist Prisma checklist. (DOC)

S1 Table Details of QUADAS-2 quality assessment for each study. This table presented the details of quality assessment for each study by the QUADAS-2 tool.
(DOC)

## References

[1] Zou Q, Wen W, Zhang XC. (2014) Presepsin as a novel sepsis biomarker. World J Emerg Med 5:16–9. 10.5847/wjem.j.1920-8642.2014.01.002 25215141 PMC4129857

[2] Dombrovskiy VY, Martin AA, Sunderram J, Paz HL. (2007) Rapid increase in hospitalization and mortality rates for severe sepsis in the United State: a trend analysis from 1993 to 2003. Crit Care Med 35:1244–50. 17414736 10.1097/01.CCM.0000261890.41311.E9

[3] Vincent JL, Rello J, Marshall J, Silva E, Anzueto A, Martin CD, et al (2009) International study of the prevalence and outcome of infection in intensive care unite. JAMA 302:2323–9. 10.1001/jama.2009.1754 19952319

[4] Walker C, Prkno A, Brunkhorst FM, Schlattmann P. (2013) Procalcitonin as a diagnostic marker for sepsis: a systematic review and meta-analysis. Lancte Infect Dis 13:426–35. 10.1016/S1473-3099(12)70323-7 23375419

[5] Balci C, Sungurtekin H, Gurses E, Sungurtekin U, Kaptanoglu B. (2003) Usefulness of procalcitonin for diagnosis of sepsis in the intensive care unit. Crit Care 7:85–90. 12617745 10.1186/cc1843 PMC154110

[6] Liu T, Chen HW, Liang DY, Hou YQ. (2014) Progress of prognostic markers in sepsis. Int J Lab Med 35:2794–6.

[7] Behnes M, Bertsch T, Lepiorz D, Lang S, Trinkmann F, Brueckmann M, et al (2014) Diagnostic and prognostic utility of soluble CD 14 subtype (presepsin) for severe sepsis and septic shock during the first week of intensive care treatment. Crit Care 18:507 10.1186/s13054-014-0507-z 25190134 PMC4174283

[8] Sandquist M, Wong HR. (2014) Biomarkers of sepsis and their potential value in diagnosis, prognosis and treatment. Clin Immunol 10:1349–1356. 10.1586/1744666X.2014.949675 PMC4654927 25142036

[9] Mussap M, Noto A, Fravega M, Fanos V. (2011) Soluble CD14 subtype presepsin (sCD14-ST) and lipopolysaccharide binding protein (LBP) in neonatal sepsis: new clinical and analytical perspectives for two old biamarkers. J Matern Fetal Neonatal Med 24:12–14. 10.3109/14767058.2011.601923 21740312

[10] Yaegashi Y, Shirakawa K, Sato N, Suzuki Y, Kojika M, Imai S, et al (2005) Evaluation of a newly identified soluble CD14 subtype as a marker for sepsis. J Infect Chemother 11:234–238. 16258819 10.1007/s10156-005-0400-4

[11] Palmiere C, Mussap M, Bardy D, Cibecchini F, Mangin P. (2013) Diagnostic value of soluble CD14 subtype (sCD14-ST) presepsin for the postmortem diagnosis of sepsis-related fatalities. Int J Legal Med 127:799–808. 10.1007/s00414-012-0804-5 23263410

[12] Vodnik T, Kaljevic G, Tadic T, Majkic-Singh N. (2013) Presepsin (sCD14-ST) in preoperative diagnosis of abdominal sepsis. Clin Chem Lab Med 51:2053–2062. 10.1515/cclm-2013-0061 23740685

[13] Gatsonis C, Paliwal P. (2006) Meta-analysis of diagnostic and screening test accuracy evaluations: methodologic primer. AJR Am J Roentgenol 187:271–281. 16861527 10.2214/AJR.06.0226

[14] Moher D, Liberati A, Tetzlaff J, Altman DG, PRISMA group. (2010) Preferred reporting items for systematic reviews and meta-analyses: the PRISMA statement. Int J Surg 8:336–341. 10.1016/j.ijsu.2010.02.007 20171303

[15] ACCP/SCCM Consensus Conference Committee. (1992) Definitions for sepsis and organ failure and guidelines for use of innovate therapies in sepsis. Crit Care Med 20:867–874.

[16] Levy MM, Fink MP, Marshall JC, Abraham E, Angus D, Cook D, et al (2003) International Sepsis Definitions Conference, 2001 SCCM/ESICM/ACCP/ATS/SIS. Intensive Care Med 29:530–538. 12664219 10.1007/s00134-003-1662-x

[17] Dellinger RP, Levy MM, Carlet JM. (2008) Surviving sepsis campaign: international guidelines for management of severe sepsis and septic shock. Crit Care Med 36: 296–327. 18158437 10.1097/01.CCM.0000298158.12101.41

[18] Whiting PF, Rutjes AW, Westwood ME, Mallett S, Deeks JJ, Reitsma JB, et al (2011) QUADAS-2: a revised tool for the quality assessment of diagnostic accuracy studies. Ann Intern Med 155:529–536. 10.7326/0003-4819-155-8-201110180-00009 22007046

[19] Zamora J, Abraira V, Muriel A, Khan K, Coomarasamy A. (2006) Meta-DiSc: a software for meta-analysis of test accuracy data. BMC Med Res Methodol 6:31 16836745 10.1186/1471-2288-6-31 PMC1552081

[20] Lijmer JG, Bossuyt PM, Heisteikamp SH. (2002) Exploring sources of heterogeneity in systematic reviews of diagnostic tests. Stat Med 21:1525–1537. 12111918 10.1002/sim.1185

[21] Mantel N, Haenszel W. (1959) Statistical aspects of the analysis of data from retrospective studies of disease. J Natl Cancer Inst 22:719–748. 13655060

[22] Walker SD. (2002) Properies of the summary receiver operating characteristic (SROC) curve for diagnostic test data. Stat Med 15:1237–1256. 10.1002/sim.1099 12111876

[23] Gatsonia C, Paliwal P. (2006) Meta-analysis of diagnostic and screening test accuracy evaluations: methodologic primer. AJR Am J Roentgenol 187:271–281. 16861527 10.2214/AJR.06.0226

[24] Dwamena B. (2007) MIDAS: Stata module for meta-analytical integration of diagnostic test accuracy studies Statistical Software Components S456880. Boston, MA: Boston College Department of Econonics.

[25] Cargnin S, Jommi C, Canonico PL, Genazzani AA, Terrazzino S. (2014) Diagnostic accuracy of HLA-B*57:01 screening for the prediction of abacavir hypersensitivity and clinical utility of the test: a meta-analysis review. Pharmacogenonics 15:963–976. 10.2217/pgs.14.52 24956250

[26] Egger M, Davey SG, Schneider M, Minder C. (1997) Bias in meta-analysis detected by a simple, graphical test. BMJ 315: 629–634. 9310563 10.1136/bmj.315.7109.629 PMC2127453

[27] Begg CB, Mazumdar M. (1994) Operating characteristics of a rank correlation test for publication bias. Biametrics 50:1088–1101. 7786990

[28] Kweon OJ, Choi JH, Park SK, Park AJ. (2014) Usefulness of presepsin (sCD14 subtype) measurements as a new marker for the diagnosis and prediction of disease severity of sepsis in the Korean population. J Crit Care 29:965–970. 10.1016/j.jcrc.2014.06.014 25042676

[29] Sargentini V, Ceccarelli G, D’Alessandro M, Collepardo D, Morelli A, D’Eqidio A, et al (2014) Presepsin as a potential marker for bacterial infection relapse in critical care patients: A preliminary study. Clin Chem Lab Med. 53:567–573. 10.1515/cclm-2014-0119 24897401

[30] Su MH, Shou ST. (2014) Prognostic value of presepsin for diagnosis and severity assessment of sepsis. Chin J Clin Lab Sci 32:106–111.

[31] Yu J, Shao Q, Wang Q, Zhang XH, Huang K. (2014) Combined determination of presepsin, procalcitonin and C reactive protein for early diagnosis and prognostic assessment of severe trauma patients with sepsis. Chin J Clin Lab Sci 32:200–203.

[32] Liu B, Chen YX, Yin Q, Zhao YZ, Li CS. (2013) Diagnostic value and prognostic evaluation of presepsin for sepsis in an emergency department. Crit Care 17:R244 10.1186/cc13070 24138799 PMC4056322

[33] Ulla M, Pizzolato E, Lucchiari M, Loiacono M, Soardo F, Forno D, et al (2013) Diagnostic and prognostic value of presepsin in the management of sepsis in the emergency department: a multicenter prospective study. Crit Care 17:R168 10.1186/cc12847 23899120 PMC4056762

[34] Shozushima T, Takahashi G, Matsumoto N, Kojika M, Okamura Y, Endo S. (2011) Usefulness of presepsin (sCD14-ST) measurement as a marker for the diagnosis and severity of sepsis that satisfied diagnostic criteria of systemic inflammatory response syndrome. J Infect Chemother 17:764–769. 10.1007/s10156-011-0254-x 21560033

[35] Shirakawa K, Naitou K, Hirose J, Takahashi T, Furusako S. (2011) Presepsin (sCD14-ST): development and evaluation of one-step ELISA with a new standard that is similar to the form of presepsin in sepsis patients. Clin Chem Lab Med 49:937–939. 10.1515/CCLM.2011.145 21345045

[36] Whiting PF, Rutjes AW, Reitsma JB, Bossuyt PM, Kleijnen J. (2003) The development of QUADAS: a tool for the quality assessment of studies of diagnostic accuracy included in systematic reviews. BMC Med Methodol 3:25. 10.1186/1471-2288-3-25 PMC305345 14606960

[37] Bates DW, Cook EF, Goldman L, Lee TH. (1990) Predicting bacteremia in hospitalized patients. A prospectively validated model. Ann Intern Med 113:495–500. 2393205 10.7326/0003-4819-113-7-495

[38] Bates DW, Sands K, Miller E, Lanker PN, Hibberd PL, Graman PS, et al (1997) Predicting bacteremia in patients with sepsis syndrome. Academic Medical Center Consortium Sepsis Preject Working Group. J Infect Dis 176:1538–1551. 9395366 10.1086/514153

[39] Endo S, Suzuki Y, Takahashi G, Shouzushima T, Ishikura H, Murai A, et al (2012) Usefulness of presepsin in the diagnosis of sepsis in a multicenter prospective study. J Infect Chmother 18: 891–897. 10.1007/s10156-012-0435-2 22692596

[40] Okamura Y, Yokoi H. (2011) Development of a point-of-care assay system for measurement of presepsin (sCD14-ST). Clin Chim Acta 412: 2157–2161. 10.1016/j.cca.2011.07.024 21839732

[41] Tang BM, Eslick GD, Craig JC, McLean AS. (2007) Accuracy of procalcitonin for sepsis diagnosis in critically ill patients: systematic review and meta-analysis. Lancet Infect Dis 7: 210–217. 17317602 10.1016/S1473-3099(07)70052-X

[42] Lee SH, Chan RC, Wu JY, Chen HW, Chang SS, Lee CC. (2013) Diagnostic value of procalcitonin for bacterial infection in elderly patients- a systemic review and meta-analysis. Int J Clin Pract 67:1350–1357. 10.1111/ijcp.12278 24246214

[43] Nismman B, Biran H, Ramu N, Heching N, Barak V, Peretz t. (2009) The diagnostic and prognostic value of ProGRP in lung cancer. Anticancer Res 29:4827–4832. 20032442

[44] Lamy PJ, Grenier J, Kramar A. (2000) Pro-gastin-releasing peptide, neuron specific enolase and chromogranin A as serum markers of small cell lung cancer. Lung Cancer 29:197–203. 10996422 10.1016/s0169-5002(00)00113-6

