# Supplementary material for: Accuracy of Presepsin in Sepsis Diagnosis: A Systematic Review and Meta-Analysis
Source: PLoS One. 2015 Jul 20;10(7):e0133057. doi: 10.1371/journal.pone.0133057 (PMC4507991; doi:10.1371/journal.pone.0133057)
Supplement: S1 Table — This table presented the details of quality assessment for each study by the QUADAS-2 tool. (DOC) [file pone.0133057.s002.doc]

**Table S1 Details of QUADAS-2 quality assessment for each study**

| Study | Risk of bias | | | | | | | | | | | Concerns of applicability | | |
| --- | --- | --- | --- | --- | --- | --- | --- | --- | --- | --- | --- | --- | --- | --- |
|  | Patient selection | | | Index test | | Reference standard | | Flow and time | | | | Patient | Index test | Reference standard |
|  | Q 1 | Q 2 | Q 3 | Q 1 | Q 2 | Q 1 | Q 2 | Q 1 | Q 2 | Q 3 | Q 4 |  |  |  |
| Behnes M (2014) | ? | ? | ☺ | ☺ | ☹ | ☺ | ☺ | ☺ | ☺ | ☺ | ☺ | ☹ | ☺ | ☺ |
| Kweon OJ (2014) | ☺ | ☺ | ☺ | ☺ | ? | ☺ | ☺ | ☺ | ☺ | ☺ | ☺ | ☹ | ☺ | ☺ |
| Sargentini V (2014) | ☺ | ☺ | ☺ | ? | ? | ☺ | ? | ☺ | ☺ | ☺ | ☺ | ☹ | ☺ | ☺ |
| Su MH (2014) | ☺ | ☹ | ☹ | ? | ☹ | ☺ | ☺ | ? | ☺ | ☺ | ☺ | ☹ | ☺ | ☺ |
| Yu J (2014) | ☺ | ☺ | ☺ | ☺ | ? | ☺ | ☺ | ☺ | ☺ | ☺ | ☺ | ☺ | ☺ | ☺ |
| Liu B (2013) | ☺ | ☹ | ☹ | ☹ | ☹ | ☺ | ? | ? | ☺ | ☺ | ☺ | ☹ | ☺ | ☺ |
| Ulla M (2013) | ☺ | ? | ? | ☺ | ☹ | ☺ | ☺ | ? | ☹ | ☹ | ☺ | ☺ | ☺ | ☺ |
| Vodnik T (2013) | ☹ | ☹ | ☹ | ☹ | ☹ | ☺ | ☹ | ? | ☺ | ☺ | ☺ | ☹ | ☺ | ☺ |
| Shozushima T (2011) | ☺ | ☹ | ☹ | ? | ☹ | ☺ | ? | ☺ | ☺ | ☺ | ☺ | ☹ | ☺ | ☺ |

Q: question; ☺: low risk; ☹: high risk; ?: unclear risk.
